# The Detection of SARS-CoV-2 Antibodies in an Exposed Human Population Is Biased by the Immunoassay Used: Implications in Serosurveillance

**DOI:** 10.3390/pathogens12111360

**Published:** 2023-11-16

**Authors:** Francisco Llorente, Elisa Pérez-Ramírez, Mayte Pérez-Olmeda, Desirée Dafouz-Bustos, Jovita Fernández-Pinero, Mercedes Martínez-Cortés, Miguel Ángel Jiménez-Clavero

**Affiliations:** 1 Centro de Investigación en Sanidad Animal (CISA-INIA), CSIC, Valdeolmos, 28130 Madrid, Spainmajimenez@inia.csic.es (M.Á.J.-C.); 2 Centro Nacional de Microbiología, Instituto de Salud Carlos III, 28220 Majadahonda, Spain; 3 Centro de Investigación Biomédica en Red de Enfermedades Infecciosas (CIBERINFEC), 28029 Madrid, Spain; 4 Madrid Salud, Madrid City Council, 28007 Madrid, Spain; 5 Centro de Investigación Biomédica en Red de Epidemiologia y Salud Pública (CIBERESP), 28029 Madrid, Spain

**Keywords:** COVID-19, SARS-CoV-2, serologic assays, ELISA, CLIA, LFA

## Abstract

The presence of SARS-CoV-2 antibodies was examined over 7 months in a population of essential service workers exposed during the first epidemic wave in Madrid (Spain). Results obtained with different serological assays were compared. Firstly, serum samples obtained in April 2020 were analyzed using eleven SARS-CoV-2 antibody detection methods, including seven ELISAs, two CLIAs and two LFAs. While all of the ELISA tests and the Roche eCLIA method showed good performance, it was poorer for the Abbott CLIA and LFA tests. Sera from 115 workers with serologically positive results in April were collected 2 and 7 months after the first sampling and were analyzed using five of the tests previously assessed. The results showed that while some ELISA tests consistently detected the presence of anti-SARS-CoV-2 antibodies even 7 months after first detection, other methods, such as the Abbott CLIA test, showed an important reduction in sensitivity for these mature antibodies. The sensitivity increased after establishing new cut-off values, calculated taking into account both recent and old infections, suggesting that an adjustment of assay parameters may improve the detection of individuals exposed to the infection.

## 1. Introduction

Since the beginning of the COVID-19 pandemic, 766 million SARS-CoV-2 infections have been reported, with almost seven million deaths [1]. In order to evaluate the real prevalence and incidence of SARS-CoV-2 exposure in different countries or regions, antibody detection assays are the commonly used methods. In response to this need, a high number of serological assays has been developed and commercialized.

These tests are designed for the detection of antibodies specific for epitopes in either the nucleocapsid (N) or the spike (S) proteins and can identify a variety of antibody isotypes (IgG, IgM, and IgA), individually or in combination [2,3]. Most of them are presented under three formats: (1) lateral flow immunoassays (LFAs), which are considered, for the most part, rapid diagnostic tests (RDTs) that can be used at the point of care (POC); (2) enzyme-linked immunosorbent assays (ELISAs); and (3) chemiluminescence immunoassays (CLIAs), including electrochemiluminescence immunoassays (eCLIAs). If the objective is to evaluate the immunity level in a given population, different methods can be used to detect neutralizing antibodies, including microneutralization assays, pseudovirus neutralization tests, and different commercially available ELISA surrogate tests [4]. The estimation of SARS-CoV-2 seroprevalence in a population is highly influenced by the performance of the different tests. Consequently, it is essential to thoroughly evaluate the sensitivity and specificity of the available serological assays to select those with better performance for use in epidemiological studies.

Several studies have compared the performance of different serological assays. Few of them, however, have used the same sample panels in their comparisons. For instance, studies carried out by the U.S. Food and Drug Administration (FDA) and the World Health Organization (WHO) used the same sample panels with all of the evaluated assays, although the number or samples was limited: 30 to 58 seropositive and 80 seronegative samples for the comparison by the FDA [5], and 199 positive and 300 negative samples for the WHO study [6]. Other studies have evaluated the performance of a wide range of tests but using data obtained by several laboratories with different sample panels [7]. In other cases, researchers used a panel with a relatively high number of samples (over one hundred), but the number of tests evaluated was low (between two and five) [8,9,10].

Antibody levels elicited from SARS-CoV-2 decline months after infection [9]. Differences in the performance of the serological assays used to detect these decreasing antibody levels will determine the number of false negative results obtained in serological surveillance [11]. The determination of the performance of serological tests in long-term studies (over 6 months) are scarce and compare few tests [9,12,13,14,15] or use a limited number of samples [11,16]. In the present stage of the pandemic, with different waves occurring around the world, people may have become infected multiple times in the past, building up a scenario where the capacity to detect both new and old infections is essential for a correct evaluation of the epidemiological situation.

In this study, we performed a head-to-head comparison of different serology tests for SARS-CoV-2 antibody detection, using samples obtained from an exposed human population two and seven months after initial detection. The evaluated assays comprise CLIA methods, broadly used in serosurveillance, ELISA tests, which do not require specialized equipment, and LFAs.

## 2. Materials and Methods

### 2.1. Serological Assays

The serological tests used in this study are described in Table 1. For the MyBiosource ELISA kit, the step involving pH adjustment of the samples indicated by the manufacturer was skipped due to insufficient sample volume.

In-house RBD ELISA assay was performed following the method previously described [17] with modifications. Briefly, 96-well plates (Maxisorp, NUNC, Roskilde, Denmark) were coated with 100 ng/well of RBD antigen, donated by the Friedrich Loeffler Institute (FLI, Greifswald—Insel Riems, Germany), in phosphate-buffered saline (PBS). Plates were incubated at 4 °C overnight. Thereafter, the plates were blocked with PBS, 0.1% (*v/v*) Tween 20 (PBST) containing 3% (*p/v*) skimmed milk, for 1 h at room temperature (RT). Sera were diluted 1:50 in PBST with 1% skimmed milk, and 100 microliters/well were incubated for 2 h at RT. Plates were washed three times with PBST and incubated with goat peroxidase conjugated anti-human IgG diluted 1:10,000 in PBST for 1 h at RT. After three additional washing steps, 100 microliters of peroxidase substrate (TMB-MAX, Neogen Corporation, Lansing, MI, USA) were added, and after 5 min of incubation in the dark at RT, the reaction was stopped with 0.5 M H_2_SO_4_. Optical density was measured at a wavelength of 450 nm using an ELISA microplate reader. The in-house NPC-2 ELISA was performed as previously described [18]. Other ELISA, CLIA, and LFA immunochromatographic assays were performed following the manufacturer’s protocols. For commercial ELISAs and CLIAs, positivity was determined using the cut-off values recommended by the manufacturers.

**Table 1:** Evaluated tests.

Manufacturer	Format	Assay Name	Target Antigen	Reference
Eurofins	ELISA	INgezim COVID 19 DR	Nucleocapsid protein	-
IDvet	ELISA	ID Screen SARS-CoV-2-N IgG Indirect	Nucleocapsid protein	-
MyBiosource	ELISA	Human COVID-19 Nucleocapsid (N) IgG/IgM ELISA kit	Nucleocapsid protein	-
Mikrogen	ELISA	RecomWell SARS-CoV-2 IgG	Nucleocapsid protein	-
BIO-RAD	ELISA	Platelia SARS-CoV-2 Virus Total Ab assay	Nucleocapsid protein	-
In-house	ELISA	NPC-2 ELISA	Nucleocapsid protein	[18]
In-house	ELISA	RBD ELISA	Spike-1 protein	[17]
Roche	eCLIA	Elecsys Anti-SARS-CoV-2 (N)	Nucleocapsid protein	-
Abbott	CLIA	Architect SARS-CoV-2 IgG	Nucleocapsid protein	-
T&D Diagnostics	LFA	2019-nCoV IgG/IgM Rapid Test	Unknown	-
Tianjin Biotechnology	LFA	COVID-19 IgG/IgM Joysbio	Unknown	-

### 2.2. Serum Samples

The first stage of this study comprised 668 serum samples collected in April 2020 in Madrid (Spain), in the midst of the first epidemic wave of COVID-19 that hit the city. The presence of the virus in Madrid was detected for the first time at the end of February, which means that SARS-CoV-2 antibodies in this population were necessarily produced by a recent (<2 months) infection. We used a subset of samples obtained from the essential services personnel participating in a previously published study [19]. These samples were obtained from policemen (48.7%), firefighters (25.4%), telecommuting workers (13.3%), burial service workers (6.4%), emergency health care workers (2.1%), and workers of other services (4%). Besides the blood samples, nasopharyngeal swabs were obtained in the same sampling day and, in some cases, also in previous days, to detect SARS-CoV-2 infection using real-time reverse-transcription polymerase chain reaction (Real Time RT-qPCR) tests. Out of 668 participants, 93 had a positive RRT-PCR result before or at the day of sample collection. The samples from these PCR-positive individuals were considered “true positives” and were used to calculate the diagnostic sensitivity of the assayed serological tests.

Participants with a positive serological result in the first stage of the study in April 2020, using Eurofins or IDvet serological assays, were invited to voluntarily donate new serum samples in June and November 2020 (2 and 7 months after the first sampling, respectively). A total of 115 participants in this second stage of the study were sampled and the sera obtained were subjected to serological analysis.

### 2.3. Statistical Analysis

To establish the optimal cut-off values for in-house NPC-2 and RBD ELISAs, optical density data were statistically analyzed using a receiver–operator characteristics (ROC) curve analysis using the GraphPad Prism 6 software. The kappa statistic, determined by the same software, was used to measure the strength of agreement between the different assays.

Due to the lack of a serological gold standard for the determination of SARS-CoV-2 specific antibodies, to perform a comparison of the performance of the different methods we used the Eurofins commercial ELISA as reference technique. This ELISA kit displays excellent sensitivity (100%) and specificity (98.2%) values [20] and is able to detect different Ig isotypes (IgM, IgG and IgA).

The sensitivity of these tests was calculated as the proportion of seropositive samples with respect to the reference method (Eurofins ELISA or samples from patients with a previous positive PCR result). Specificity was calculated as the proportion of seronegative samples with respect to the reference ELISA.

## 3. Results

### 3.1. Comparative Performance of Different Serological Assays for Detection of Recent Infections

In the first step of this study, the 668 samples collected in April 2020 were analyzed using Eurofins ELISA and T&D LFA tests. Then, a representative subset of these samples (ranging from 167 to 416) with a proportional distribution of positive and negative sera was also analyzed with six additional ELISA tests, two CLIAs, and one additional LFA test. The results obtained with the in-house NPC-2 ELISA have been previously published [18].

The ROC analysis comparing the results obtained with the RBD and reference ELISA in this first stage of the study indicated that the area under the curve (AUC) was 0.9805 (95% confidence interval (CI): 0.9679–0.9930), showing a high diagnostic accuracy for the RBD ELISA. Based on the ROC curve, a cut-off value of 3.5 (OD sample/OD negative control) was set. For the in-house NPC2 ELISA, we used a cut-off value of four, as determined in a previous study [18].

The sensitivity and specificity values of the assayed tests ranged from 89.5% to 99.3% and from 75.9% to 97.3%, respectively (Table 2). All ELISA tests showed similar sensitivity values (ranging from 95.4% for in-house RBD to 99.3% for Mikrogen). Specificity values ranged from 91.6% for Mikrogen to 97.3% for BIO-RAD. The two assayed CLIA tests showed important differences in their sensitivity, with a lower sensitivity observed in the Abbott CLIA (89.5%) as compared to Roche CLIA (99.0%), although in terms of specificity, both showed similar results (96.3% for Roche and 96.7% for Abbott). LFA tests displayed a lower performance, at least in relation to specificity, as compared with ELISA tests. In the case of the T&D LFA, we obtained sensitivity and specificity values of 94.8% and 92.0%. For the Tianjin LFA, two batches were assayed with differing results, obtaining sensitivity values of 89.9% in batch A and 99.1% in batch B, and specificity values of 90.8% in batch A and 75% in batch B. In view of this relevant variation, we used two additional batches of this test to analyze a subset of samples and we obtained intermediate results.

The outcomes of the kappa analysis performed to evaluate the agreement of results between serological tests are shown in Table 3. The highest correlations were observed between all ELISAs and the Roche eCLIA, showing an almost perfect agreement (>0.8) with values over 0.85. A lower correlation with the ELISAs was observed for the Abbott CLIA, with values between 0.76 and 0.8, which correspond to “substantial agreement” (0.61–0.8) [21]. The two tested LFAs displayed the lowest correlation values when compared with both ELISAs and CLIA tests. Likewise, a limited concordance was obtained when both LFA tests were compared.

In a further stage, we aimed to determine the diagnostic sensitivity of the assays. For this purpose, we compared the results obtained with the different tests when applied to a subset of “true positive” samples that were obtained from PCR-positive individuals. Diagnostic sensitivity values of the different tests are shown in Table 4. Considering the cut-off values previously described, the diagnostic sensitivity for ELISA tests ranged from 90.3% in NPC-2 to 93.5% in Mikrogen, BIO-RAD and RBD. For CLIA tests, Abbott Architect showed a lower sensitivity (88.8%) than ROCHE Elecsys (95.5%). The diagnostic sensitivity of the T&D LFA was 87.1%, while for the Tianjin Joysbio LFA, it ranged from 81.7 to 96.8% (depending on the batch). ELISA tests and Roche CLIA, which showed the highest correlation agreement in the previous kappa analysis, also displayed the highest diagnostic sensitivities (over 90%) when analyzing “true positive” samples. The values obtained for diagnostic sensitivity were slightly lower than those observed when comparing the different assays with the reference ELISA test.

### 3.2. Comparative Performance of Serological Assays at Different Times Post-Infection

The performance of five ELISA tests and an Abbott Architect CLIA was evaluated at different times after the first sampling. For that purpose, 115 seropositive patients (based on Eurofins and/or IDvet results) voluntarily participated in a second stage of the study. Sera from these patients, obtained 2 and 7 months after their initial diagnosis, were subjected to analysis. For the sera collected two months after the first sampling, the positivity rate was similar to the initial one for Eurofins, IDvet, BIO-RAD, and RBD ELISA tests, whereas a decrease in sensitivity was observed for the in-house NPC-2 ELISA (15% reduction) and Abbott CLIA (5% reduction) methods. Antibody detection at 7 months post-initial sampling was still highly similar for Eurofins and BIO-RAD methods but important limitations in sensitivity were observed for the other methods, with reduced positivity rates ranging from 43% for the Abbot CLIA to 72% for the IdVet ELISA (Table 5).

New cut-off values were calculated for in-house ELISAs using a receiver–operator characteristics (ROC) curve analysis including data obtained from these tests in April and those obtained in June and November. For the NPC-2 ELISA, the area under the curve (AUC) was 0.9649 (95% confidence interval (CI): 0.9500–0.9798) (*p* < 0.0001), showing a high diagnostic accuracy. Based on the ROC curve, a new cut-off value of 2.5 (OD sample/OD negative control) was set for this ELISA. Similarly, for the RBD ELISA, the area under the curve (AUC) was 0.9574 (95% confidence interval (CI): 0.9403–0.9745) (*p* < 0.0001) and a cut-off of 2.5 was set.

With the initial cut-off values, the sensitivity and specificity rates of the NPC-2 ELISA were 79.7% and 96.5%, respectively. If we apply the new cut-off value, the sensitivity increases to 90.2% but specificity decreases to 91.7%. For the RBD ELISA, the initial sensitivity and specificity rates were 85.9% and 92.9%, respectively, while with the new cut-off value, these rates were 92.5% and 89.8%.

Considering these new cut-off values, the sensitivity rate of the in-house ELISAs moderately increased both in the first sampling (Table 2) and when considering the subset of sera from PCR-positive patients (Table 4). The specificity values decreased in relation to the reference ELISA, but remained above 90% (Table 2). Using the new cut-off values in the analysis of sera obtained seven months after the first sampling, the sensitivity increased by 20% for the NPC-2 and 15% for the RBD ELISA, reaching values similar or even better than those obtained with the commercial IDvet ELISA test (Table 5).

When the analysis was restricted to the “true positive” samples, the results were highly similar to those observed when considering all seropositive sera. The highest positivity rates 7 months after the first seropositive results were observed with Eurofins and BIO-RAD ELISA tests, and the lowest was observed with the Abbott Architect CLIA (Table 6).

Additionally, 50 samples collected in April and June were analyzed to assess batch-to-batch variations in the Tianjin LFA test. For that purpose, the two batches previously used in the first stage of the study were compared. Differences between these batches increased when analyzing sera obtained two months after the first sampling. When using the batch with the highest sensitivity in April (batch B), the results in June did not show a reduction in sensitivity for IgG antibodies; but a remarkable reduction in sensitivity, from 80% to 48%, was observed for IgM antibodies. With batch A, a reduction in sensitivity was observed in June both for IgG and IgM antibodies (Table 7).

## 4. Discussion

The present study aimed to determine the performance of six serological tests when applied to sera obtained at different times post infection. For that, we used sera obtained from a cohort of SARS-CoV-2-infected individuals 2 and 7 months after the first antibody detection. The tests used in this follow-up study were selected based on the results of a previous performance analysis where 11 serological tests were evaluated using serum samples obtained in the first months of the pandemic.

This first stage of the study (performance analysis of 11 serological tests) included a high number of samples (668), obtained from essential service personnel working for the Madrid City Council (Spain), professionally exposed to the infection during the first wave of the pandemic. The cohort included a high percentage of either asymptomatic or mildly symptomatic individuals [19], this being a better estimation for the situation of the general population than cohorts composed of patients admitted to hospitals with a higher percentage of clinically severe manifestations, which are the kind most often analyzed in serological studies of this type [22].

To cover the different available options for serological diagnosis, the assays analyzed in this work included some of the most broadly used commercially available ELISA tests; two in-house ELISA tests; two CLIA methods, frequently used in broad-scale serosurveillance studies; and two LFAs, which can be considered as POC methods. We did not include in this study tests that specifically detect neutralizing antibodies because our objective was not the determination of the immunity level of the population but rather the detection of previous infection, and this includes the presence of both neutralizing and non-neutralizing antibodies.

The results obtained with the 11 assayed serological tests confirmed that the assays with higher correlation rates were those with higher diagnostic sensitivity (the capacity to detect specific antibodies in PCR-confirmed infected individuals). The best diagnostic performance, when considering recent infections, was displayed by the ELISA tests and Roche eCLIA test, with sensitivity values over 95% and specificity values over 90% (Table 2 and Table 4) also showing high agreement rates among them (Table 3). In contrast, the Abbott CLIA showed a lower sensitivity (89.5%). This sensitivity value is within the range of values observed in other studies where this CLIA was used (from 38.8% to 97.9%) [10], with values under 90% in most cases.

While the results obtained with all the ELISA tests showed a high correlation, the two assayed CLIAs directed against the N protein showed a lower correlation between one another, with better specificity and sensitivity results for ROCHE Elecsys than for Abbott Architect. This different performance was previously observed in sera collected from SARS-CoV-2 infected patients in the first 8 weeks after RT-PCR detection [23].

Sensitivity and specificity values of the assayed tests varied depending on the reference method used. If only sera from PCR-positive individuals were considered, the sensitivity rates were slightly lower than those obtained using the Eurofins ELISA as reference technique. This difference may be due to the fact that sera from PCR-positive individuals might lack detectable antibodies in some instances, e.g., at the early acute stage of the infection [7]. In fact, two samples from PCR-positive individuals gave consistently seronegative results in all of the serological assays and one sample was only detected with a weak signal by the Tianjin Joysbio LFA test. Both LFAs performed less efficiently when compared to the ELISA and CLIA methods. Moreover, large intra-batch differences in test performance were observed in at least one of the LFAs (Tianjin). In more detail, the batch with the highest sensitivity suffered from an important decrease in specificity. Although LFAs can be extremely useful as POC methods due to their easy use and quick results, the risk of variability between batches must be taken into account when using them in serological surveillance, and appropriate batch control procedures should be put in place to avoid such risks of variation even when using the same assay.

Different studies have suggested better sensitivity and specificity values for CLIAs than for ELISA methods [24]. However, in this study, we detected sensitivity and specificity values in different ELISAs that were similar to those observed when using the Roche CLIA. Furthermore, sensitivity and specificity values were higher for the assayed ELISAs than for the Abbott CLIA. These results indicate that the performance of ELISA tests for SARS-CoV-2 serological surveillance can be as good as that of CLIA methods, or even better.

In regards to the antigens used in the serological tests studied, most of them (six out of seven ELISAs and both CLIAs) are directed against the nucleoprotein (N) and only one is directed against the S protein (the in-house RBD ELISA). In the current epidemiological situation, with a high percentage of the population having been immunized with S protein-based vaccines, the serological methods that use the N protein as an antigen are the most appropriate to determine the actual incidence of the infection. Additionally, for a differential detection of natural and vaccine-induced antibody responses, a protocol with two assays, one directed against the N protein and another one against the S protein (as RBD ELISA), would be useful.

For the prospective study using sera collected 2 and 7 months after the first sampling, we selected three broadly used ELISA tests, which have shown a good performance in this study and other studies for recently infected patients [5,6,7,20,25]; two in-house ELISA tests [17,18]; and one CLIA method broadly used in serosurveillance studies [26,27,28]. A relevant variability was observed in the results obtained with the different methods assayed. While some of the commercial ELISA tests (Eurofins and BIO-RAD) had a high level of positivity 7 months after the first serological detection, other methods, such as the Abbott CLIA, showed an important reduction in the detection of seropositive samples. Considering these results, although ELISA tests are medium-throughput methods and CLIA are high-throughput methods, commercially available ELISAs seem more effective than certain CLIA methods that have been frequently used in serosurveillance studies. This is highly relevant, since laboratories lacking the complex and expensive equipment required for CLIA analysis can perform efficient serological surveillance using ELISA tests. The detection capacity of these ELISAs is equivalent for recent infections, and even better for old infections, than that displayed by some widely used CLIA tests such as the Abbott Architect.

Although the Roche CLIA was not included in this long-term antibody detection study, other researchers have confirmed that the Roche Elecsys method is more efficient than the Abbott Architect for the detection of antibodies a long time after infection [11]. Therefore, the Roche method seems to be a better diagnostic tool for serosurveillance in populations with a high percentage of old infections. The percentage of positivity for the Roche CLIA seven months after first seropositive result determined in these previous studies [11] is similar to that observed with the best performing methods in our study: Eurofins and BIO-RAD ELISAs.

Different aspects can affect test performance, and consequently their sensitivity, at different times post-infection. These aspects may include the antibody isotype detected, the antigen used and the methodology of detection [29]. In this work, we detected a much lower decrease in sensitivity for competitive assays (Eurofins and BIORAD) than for indirect assays (IDvet and in-house ELISAs and Abbott CLIA).

The LFA test could only be assayed with sera collected 2 months after the first detection. These results revealed severe variability in the performance between batches, with sharp or smooth declines in IgG detection depending on the batch used. As expected, two months after the first detection, IgM antibody levels were much lower than IgG levels.

When the first assays for SARS-CoV-2 antibody detection were developed, they were validated using the available sera obtained during the first COVID-19 wave, hence that positive sera corresponded to recent infections. Thus, the cut-off values established at that time were based only on this type of sample. Nevertheless, the selected cut-off values with only recently infected sera might be less effective when the proportion of old infections increases in the population. After several infection waves, the initially established cut-off values should be updated by taking into account the different antibody responses derived from both recent and old infections. In this study, we confirmed that the use of these new cut-off values in the in-house ELISA tests increased their sensitivity for old infections (7 months after first detection) to levels similar or higher than those obtained with the commercial IDvet ELISA test, while specificity decreased very slightly. In the current epidemiological situation, a minor reduction in specificity can be acceptable provided that a relevant increase in sensitivity is achieved. A study performed including a cohort of mild/asymptomatic infected individuals that were serologically analyzed 8 months after infection [13] also describes divergences of sensitivity for three different serological tests, suggesting that an adjustment for the time-varying sensitivity of the assay is required to avoid an underestimation of the true number of seropositive individuals.

Regarding the in-house methods studied, NPC-2 and RBD ELISAs, the results obtained indicate that there is room for improvement in both cases. These methods are indirect ELISAs, designed for the specific detection of IgG. Improved assays may be developed using the same antigens, in formats such as double recognition (DR) or competition ELISAs, which may allow for increased sensitivity and specificity and the detection of other Ig subtypes.

Another aspect to consider is the increasing number of viral variants. In this work, we used samples from the beginning of the pandemic, when the level of viral variation was very low and only early (pre-Alpha variant) lineages were circulating in Spain [30]. In the current context, a validation of the different assays using sera from patients infected with new variants can be advisable.

The estimation of cumulative infection in previous months is affected by the performance of the serological tests, with the infection levels of older viral waves severely underestimated due to the observed low sensitivity of different serological assays. The current epidemiological situation is becoming complex, with different waves inducing a mix of recent and past infections and a high percentage of the population being vaccinated. Consequently, for a more accurate evaluation of the proportion of population previously infected by SARS-CoV-2, and a better tracking of the evolution of the pandemic, an appropriate assay selection and performance adjustment, including an update of cut-off values, is required.

## Figures and Tables

**Table 2:** Results obtained from sera collected in the first sampling (April) for ELISA, CLIA and LFA tests. Sensitivity and specificity values were calculated considering the Eurofins ELISA as a reference technique.

	N	IgM		IgG			Total Igs ^1^			Sensitivity % (95% CI)	Specificity % (95% CI)
		Positive	Negative	Positive	Borderline/Doubtful	Negative	Positive	Borderline/Doubtful	Negative		
ELISA											
Eurofins	668	-	-	-	-	-	194	8	466	-	-
IDvet	414	-	-	199	8	207	-	-	-	97.9 (94.8–99.4)	95.9 (92.4–98.1)
MyBiosource	171	-	-	-	-	-	90	-	81	97.8 (92.1–99.7)	96.7 (90.6–99.3)
Mikrogen	256	-	-	146	3	107	-	-	-	99.3 (96.0–100)	91.6 (85.1–95.9)
BIO-RAD	414	-	-	-	-	-	193	5	216	96.4 (92.7–98.5)	97.3 (94.2–99.0)
In-house NPC-2 (cut-off 4) ^2^	416	-	-	194	-	222	-	-	-	96.4 (92.7–98.5)	96.4 (93.1–98.4)
In-house NPC-2 (cut-off 2.5) *	416	-	-	210	-	206	-	-	-	99.0 (96.3–99.9)	91.9 (87.5–95.2)
In-house RBD (cut-off 3.5)	415	-	-	199	-	216	-	-	-	95.4 (91.4–97.9)	93.7 (89.6–96.5)
In-house RBD (cut-off 2.5) *	415	-	-	212	-	203	-	-	-	98.5 (95.6–99.7)	90.5 (85.9–94.0)
CLIA											
Roche	404	-	-	-	-	-	193	5	216	99.0 (96.3–100)	96.3 (92.7–98.4)
Abbott	404	-	-	178	-	226	-	-		89.5 (84.3–93.5)	96.7 (93.4–99.0)
LFA											
T&D	668	65	603	199	-	469	222	-	446	94.8 (90.7–97.5)	92.0 (89.2–94.3)
Tianjin (batch A)	257	16	241	133	-	124	134	-	123	89.8 (83.4–94.3)	90.8 (84.2–95.3)
Tianjin (batch B)	167	96	71	121	-	46	125	-	42	99.1 (95.2–100)	75.9 (62.4–86.5)

For the determination of specificity and sensitivity, borderline/doubtful results were considered as negative. ^1^ Total Igs for Eurofins and BIO-RAD ELISAs; IgG and IgM for MyBiosource, and either IgM and/or IgG for LFAs tests. ^2^ Data from an in-house NPC-2 ELISA previously published [18]. * Cut-off values obtained using an ROC curve analysis with all analyzed samples (obtained in the first and second stage of the study).

**Table 3:** Agreement of results between serological tests based on kappa values. Values with an almost perfect agreement (>0.8) are indicated in grey.

BIO-RAD	0.91										
MyBiosource	0.92	0.97									
Mikrogen	0.86	0.87	0.92								
IDvet	0.90	0.88	0.86	0.87							
RBD	0.87	0.87	0.89	0.88	0.91						
NPC-2	0.93	0.90	0.87	0.88	0.95	0.87					
Roche	0.92	0.92	0.94	0.90	0.94	0.92	0.91				
Abbott	0.83	0.85	0.76	0.78	0.84	0.83	0.90	0.85			
T&D	0.79	0.80	0.78	0.78	0.83	0.78	0.82	0.82	0.76		
Tianjin A	0.75	0.78	0.82	0.76	0.75	0.79	0.81	0.79	0.76	0.78	
Tianjin B	0.80	0.78	0.84	0.83	0.84	0.86	0.81	0.86	0.74	0.76	0.71
	Eurofins	BIO-RAD	MyBiosource	Mikrogen	IDvet	RBD	NPC-2	Roche	Abbott	T&D	Tianjin A

**Table 4:** Diagnostic sensitivity of the different assays calculated as the percentage of seropositive results obtained in “true positive” patients (SARS-CoV-2 PCR-positive patients).

	Number of Seropositive Individuals/Number of Individuals Tested (%) (95% CI)				
	Days Post-PCR Detection				
	<7 d	7–14 d	15–21 d	>21 d	All Sera
**ELISAs**					
Eurofins	53/56 (94.6) (85.1–98.9)	21/22 (95.5) (77.2–99.9)	19/20 (95.0) (75.1–99.9)	12/14 (85.7) (57.2–98.2)	86/93 (92.5) (85.1–96.9)
IDvet	53/56 (94.6) (85.1–98.9)	21/22 (95.5) (77.2–99.9)	18/20 (90.0) (81.5–100)	12/14 (85.7) (57.2–98.2)	85/93 (91.4) (83.8–96.2)
MyBiosource	24/28 (85.7) (67.3– 96.0)	14/14 (100) (76.8–100)	3/3 (100) (29.2–100)	7/7 (100) (59.0–100)	38/42 (90.5) (77.4–97.3)
Mikrogen	53/56 (94.6) (85.1–98.9)	21/22 (95.5) (77.2–99.9)	19/20 (95.0) (75.1–99.9)	13/14 (92.9) (66.1–99.8)	87/93 (93.5) (86.5–97.6)
BIO-RAD	52/56 (92.9) (82.7–98.0)	22/22 (100) (84.6–100)	19/20 (95.0) (75.1–99.9)	13/14 (92.9) (66.1–99.8)	87/93 (93.5) (86.5–97.6)
In-house NPC-2 (cut-off 4) ^1^	53/56 (94.6) (85.1–98.9)	20/22 (90.9) (70.8–98.9)	18/20 (90.0) (81.5–100)	12/14 (85.7) (57.2–98.2)	84/93 (90.3) (82.4–95.5)
In-house NPC-2 (cut-off 2.5) *	53/56 (94.6) (85.4–98.9)	21/22 (95.5) (77.2–99.9)	20/20 (100) (83.2–100)	12/14 (85.7) (57.2–98.2)	87/93 (93.5) (86.5–97.6)
In-house RBD (cut-off 3.5)	51/56 (91.1) (80.4–97.0)	20/22 (90.9) (70.8–98.9)	20/20 (100) (83.2–100)	14/14 (100) (76.8–100)	87/93 (93.5) (86.5–97.6)
In-house RBD (cut-off 2.5) *	55/57 (96.5) (87.9–97.6)	21/22 (95.5) (77.2–99.9)	20/20 (100) (83.2–100)	14/14 (100) (76.8–100)	90/93 (96.8) (90.9–99.3)
**CLIA**					
Roche	52/54 (96.3) (87.3–99.5)	21/21 (100) (83.9–100)	19/20 (95.0) (75.1–99.9)	11/12 (91.7) (61.5–99.8)	85/89 (95.5) (88.9–98.8)
Abbott	50/54 (92.6) (82.1–97.9)	18/21 (85.7) (63.7–97.0)	18/20 (90.0) (81.5–100)	11/12 (91.7) (61.5–99.8)	79/89 (88.8) (80.3–94.5)
**LFAs**					
T&D	51/56 (91.1) (80.4–97.0)	19/22 (86.4) (65.1–97.1)	18/20 (90.0) (81.5–100)	12/14 (85.7) (57.2–98.2)	81/93 (87.1) (78.5–93.2)
Tianjin (batch A)	46/56 (82.1) (69.6–91.1)	19/22 (86.4) (65.1–97.1)	16/20 (80.0) (56.3–94.3)	12/14 (85.7) (57.2–98.2)	76/93 (81.7) (72.4–89.0)
Tianjin (batch B)	54/56 (96.4) (87.7–99.6)	21/22 (95.5) (77.2–99.9)	20/20 (100) (83.2–100)	14/14 (100) (76.8–100)	90/93 (96.8) (90.9–99.3)

Serum samples obtained from workers with a positive PCR result at different days before serum sampling are included in more than one column of days post-PCR detection. For LFAs, either IgM- and/or IgG-positive results have been considered as “seropositive”. ^1^ Data from an in-house NPC-2 ELISA previously published in Williams et al. [18]. * Cut-off values obtained using an ROC curve analysis with all available samples (from the first and second stages of the study).

**Table 5:** Percentage of seropositive samples collected at different times (June and November 2020) in patients identified as seropositive in April, as determined using Eurofins and/or IDvet ELISAs (*n* = 115).

	Number of Seropositive Samples (%)		
	April	June	November
Eurofins	112 (97%)	113 (98%)	113 (98%)
IDvet	114 (99%)	112 (97%)	83 (72%)
BIO-RAD	109 (95%)	113 (98%)	107 (93%)
In-house NPC-2 (cut-off 4)	112 (97%)	94 (82%)	58 (50%)
In-house NPC-2 (cut-off 2.5) *	114 (99%)	106 (92%)	81 (70%)
In-house RBD (cut-off 3.5)	109 (95%)	103 (90%)	75 (65%)
In-house RBD (cut-off 2.5) *	113 (98%)	108 (94%)	92 (80%)
Abbott	106 (92%)	100 (87%)	50 (43%)

* Cut-off values obtained using an ROC curve analysis with all available samples (from the first and second stage of the study).

**Table 6:** Percentage of seropositive samples collected at different times (June and November 2020) in patients identified as seropositive in April, as determined using Eurofins and/or IDvet ELISAs, and with a previous positive PCR result (*n* = 52).

	Number of Seropositive Samples (%)		
	April	June	November
Eurofins	52 (100%)	51 (98%)	51 (98%)
IDvet	52 (100%)	51 (98%)	40 (77%)
BIO-RAD	51 (98%)	51 (98%)	48 (98%)
In-house NPC-2 (cut-off 4)	52 (100%)	46 (89%)	28 (54%)
In-house NPC-2 (cut-off 2.5) *	52 (100%)	50 (96%)	41 (79%)
In-house RBD (cut-off 3.5)	49 (96%)	49 (96%)	41 (79%)
In-house RBD (cut-off 2.5) *	52 (100%)	51 (98%)	46 (89%)
Abbott	50 (96%)	46 (88%)	24 (46%)

* Cut-off values obtained using an ROC curve analysis with all available samples (from the first and second stage of the study).

**Table 7:** Results obtained from the Tianjin B. Joysbio LFA assay in serum samples from serologically positive patients, as determined using Eurofins and/or IDvet ELISAs in April (*n* = 50).

		Number of Seropositive Samples (%)	
		April	June
Batch A	IgM	6 (12%)	0 (0%)
	IgG	46 (92%)	33 (66%)
	Total Igs	46 (92%)	33 (66%)
Batch B	IgM	40 (80%)	24 (48%)
	IgG	50 (100%)	50 (100%)
	Total Igs	50 (100%)	50 (100%)

## Data Availability

Data are available upon reasonable request to the authors.
